# Daily light exposure profiles and the association with objective sleep quality in patients with Parkinson’s disease: The PHASE study

**DOI:** 10.1093/sleep/zsae036

**Published:** 2024-02-08

**Authors:** Kenji Obayashi, Keigo Saeki, Yoshiaki Tai, Yuki Yamagami, Yuichi Esaki, Tadanobu Yoshikawa, Kazuma Sugie, Hiroshi Kataoka

**Affiliations:** Department of Epidemiology, Nara Medical University School of Medicine, Nara, Japan; Department of Epidemiology, Nara Medical University School of Medicine, Nara, Japan; Department of Epidemiology, Nara Medical University School of Medicine, Nara, Japan; Department of Epidemiology, Nara Medical University School of Medicine, Nara, Japan; Department of Psychiatry, Fujita Medical University School of Medicine, Aichi, Japan; Department of Ophthalmology, Nara Medical University School of Medicine, Nara, Japan; Department of Neurology, Nara Medical University School of Medicine, Nara, Japan; Department of Neurology, Nara Medical University School of Medicine, Nara, Japan

**Keywords:** light exposure, Parkinson’s disease, sleep, circadian, actigraphy

## Abstract

**Study Objectives:**

Light information crucially influences sleep initiation and continuity. The purpose of this study was to compare daily light exposure between patients with Parkinson’s disease (PD) and non-PD older adults and evaluate the association of daily light exposure with objective sleep measures in patients with PD.

**Methods:**

In this cross-sectional study of 189 outpatients with PD and 1101 community-dwelling older adults (controls), daily light exposure was measured using wrist light meters during the daytime and light meters set in the bedrooms during the nighttime, and objective sleep quality was measured by wrist actigraphy.

**Results:**

The median duration of exposure to ≥ 1000 lux light was significantly shorter in patients with PD than in controls. The median nighttime light intensity was higher in patients with PD than in controls. Among patients with PD, multivariable analysis suggested that the highest quartile of exposure to ≥ 1000 lux light during the daytime was linked to significantly higher sleep efficiency (SE) by 8.0% and shorter wake after sleep onset (WASO) by 36.9 minutes than the lowest quartile. During the nighttime, the highest quartile of mean light intensity had significantly lower SE by 6.8%, longer WASO by 24.1 minutes, longer sleep onset latency, and higher fragmentation index, than the lowest quartile. Importantly, daytime and nighttime light levels were independently associated with objective sleep measures.

**Conclusions:**

The present study illustrated that greater daytime light exposure and lower nighttime light exposure are significantly associated with better objective sleep measures in patients with PD.

## Graphical Abstract

**Graphical Abstract:**
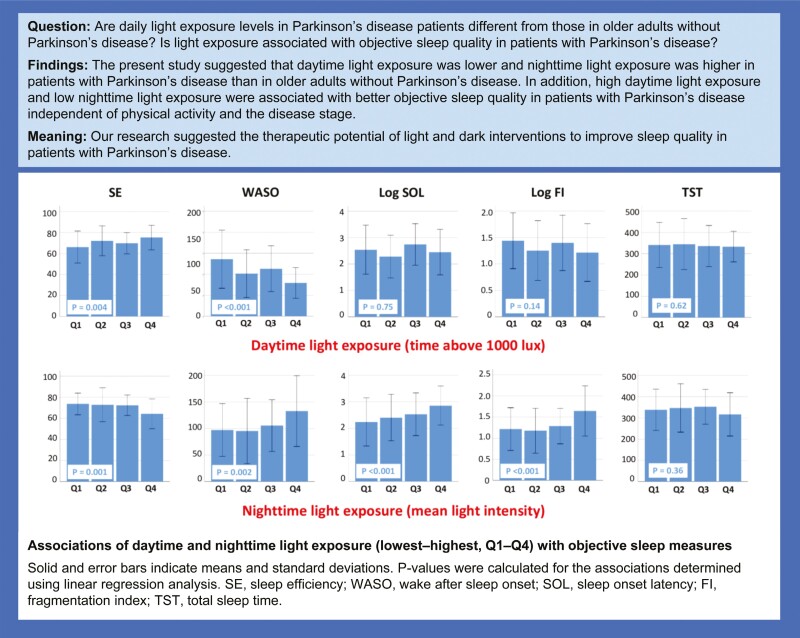

**Statement of Significance**

The present cross-sectional study suggested that patients with Parkinson’s disease have lower daytime light exposure and higher nighttime light exposure than older adults without Parkinson’s disease, and among patients with Parkinson’s disease, greater daytime light exposure and lower nighttime light exposure were significantly associated with better objective sleep measures independent of potential confounders, including daytime physical activity and disease stage. Importantly, daytime and nighttime light exposure was independently associated with objective sleep measures. Further studies are warranted to explore the nature of this observation.

## Introduction

Sleep–wake cycles are behavioral and physiological processes controlled by the circadian timing system in the suprachiasmatic nucleus (SCN) of the hypothalamus [1]. Light information crucially influences this system, and light exposure inappropriately timed against the solar cycle can desynchronize the internal biological rhythms, resulting in circadian misalignment [2]. Previous studies performed under controlled laboratory conditions have shown that the daytime and nighttime inputs of light information into the SCN can change the core body temperature, melatonin secretion, and brain activity [3–5]. In a recent systematic review of epidemiological studies, sleep quality was affected by light exposure at night (LAN) [6].

Parkinson’s disease (PD) is a major cause of disability globally [7]. Patients with PD frequently experience sleep problems related to the initiation and maintenance of sleep [8, 9]. Neurodegeneration of the sleep regulatory area is a potential cause of sleep problems in patients with PD. Notably, circadian misalignment, which is prevalent in PD, is another major cause of sleep problems [10]. A recent systematic review and meta-analysis of two randomized controlled trials suggested no significant changes in sleep quality following bright light therapy in patients with PD [11]. These studies exposed patients to 10 000 lux bright light in the morning and evening, but evening exposure to bright light causes melatonin suppression and circadian phase delay, possibly resulting in decreased sleep quality [12–16]. Thus, the associations of daytime and nighttime light exposure with sleep initiation and continuity remain uncertain. In addition, daytime and nighttime light exposure among patients with PD in real-life settings remains unclear.

The purpose of this study was to compare daily light exposure between patients with PD and non-PD older adults and evaluate the association of daily light exposure with objective sleep measures, especially in sleep initiation and continuity in patients with PD.

## Materials and Methods

### Study participants

We enrolled 202 outpatients with PD in the “Parkinson’s disease and the relationships with circadian biological rhythms and sleep” (PHASE) study at Nara Medical University Hospital and its related hospitals between October 2014 and April 2017 [17]. PD was diagnosed using the UK Parkinson’s disease Society Brain Bank criteria [18]. Among the 202 outpatients, 189 successfully completed actigraphic and bedroom light assessments, maintained a sleep diary for 7 days consecutively, and underwent basic neurologic examinations. All patients were administered stable medication doses for at least 1 month before enrollment. None of the patients exhibited multiple-system atrophy, progressive supranuclear palsy, dementia with Lewy bodies, or any other atypical parkinsonian syndrome such as vascular Parkinsonism. Moreover, no patient presented large vessel disease, cerebral infarction, brain tumor, or a history of cranial surgical intervention, including deep brain stimulation surgery.

Individuals residing in the community were selected from the “Housing environments and health investigation among Japanese older people in Nara, Kansai region: a prospective community-based cohort” (HEIJO-KYO) study to form the control group [19]. A baseline survey was conducted between September 2010 and April 2014 for 1127 individuals aged ≥ 60 years. We excluded controls diagnosed with PD and those who did not complete the home actigraphy measurements, resulting in 1101 remaining participants. The study protocol was approved by the Ethics Committee of Nara Medical University, and all participants provided written informed consent.

### Measurement of daily light exposure

Daytime light exposure from rising time to bedtime was measured in 1-minute intervals using a wrist light meter (Actiwatch 2; Respironics Inc., Murrysville, PA, USA) worn on the non-dominant wrist for over 7 days in patients with PD and 2 days in controls. The device includes a photodiode with a spectral sensitivity similar to that of the human eye (luminance sensitivity, 0–150 000 lux). Special rubber bands were used to prevent interference with the participants’ shirtsleeves. Daytime values < 1 lux were excluded from the data calculations, as they were considered artifact data due to sensor coverage. If more than half of the daytime period light data were interpreted as artifacts, they were declared missing. The duration of exposure to light exceeding 1000 lux in intensity during the daytime was measured.

Nighttime light exposure from bedtime to rising time was measured in < 2-minute intervals using portable light meters (TR-74Ui [luminance sensitivity, 0–130 000 lux; accuracy, ±5%]; T&D Corp. Nagano, Japan; and LX-28SD [luminance sensitivity, 0–100 000 lux; accuracy, ±4%]; Sato Shouji Inc., Kanagawa, Japan) for seven nights in patients with PD and two nights in controls. The light meters were different between PD and controls (PD, TR-74Ui; and controls, LX-28SD), and these two light meters were not validated. The devices were placed facing the ceiling 60 cm above the floor at the head of the participant’s bed. The light meter and wrist light meter measurements were synchronized. The time above the threshold intensity of 10 lux in minutes was determined to reflect the value with minimal effects on human physiology.

### Measurements of objective sleep quality

Physical activity was measured in 1-minute intervals using Actiwatch 2 worn on the non-dominant wrist for seven nights for patients with and two nights for controls. To assess the sleep quality, the participants used standardized sleep diaries to report the clock time of their bedtime and rising time. Actiware version 5.5 software (Respironics Inc.) automatically determined the sleep status in each epoch, sleep onset, and sleep termination, using the specified algorithm. Awake epochs were defined as > 10 activity counts and > 40 counts/min for patients with PD and controls, respectively, because lower activity thresholds have better correlations with polysomnographic data in patients with PD [20]. Sleep onset was defined as the initial minute of immobility followed by immobility lasting at least 5 minutes in patients with PD and 10 minutes in controls. Similarly, sleep termination in patients with PD and controls was defined as the last minute after a 5-minute and 10-minute period of immobility, respectively.

The following five objective sleep parameters were determined from the actigraphic and self-reported bedtime and rising time data:

- Sleep efficiency (SE): the percentage of sleeping time relative to the total time between bedtime and rising time.
- Wake after sleep onset (WASO): the time spent awake between sleep onset and offset.
- Sleep onset latency (SOL): the time between bedtime and sleep onset.
- Total sleep time: the time between bedtime and rising time × SE.
- Fragmentation index (FI): the percentage of the number of 1-minute epochs scored as immobile divided by the total number of epochs scored as immobile during the time in bed.

### Other variables

Body weight and height were used to compute the body mass index (BMI). Furthermore, a self-administered questionnaire was used to record smoking, drinking, and socioeconomic information. Based on the participants’ current therapies, hypertension, and diabetes diagnoses were recorded. The sleep medications included benzodiazepine receptor agonists, non-benzodiazepine hypnotics, melatonin receptor agonists, and orexin antagonists. A standardized urination diary was used to log the nocturnal void frequency, excluding the last void at bedtime and the first-morning void. Actiwatch 2 was used to record the daytime physical activity at 1-minute intervals. The day length (sunrise to sunset) was determined based on the first day of measurements in Nara, Japan, from the National Astronomical Observatory of Japan website. PD stage in the “ON” state was categorized according to the Hoehn–Yahr staging system as early (I–II) or late (III–V) [21]. The daily dose of anti-parkinsonian agents during the start of the study was converted to the levodopa equivalent dose as follows [22]: (regular levodopa dose × 1) + (levodopa-controlled release dose × 0.75) + (entacapone or Stalevo dose × 0.33) + (pramipexole dose × 100) + (ropinirole dose × 20) + (rotigotine dose × 30) + (pergolide and cabergoline doses × 67) + (bromocriptine dose × 10) + (selegiline dose × 10) + (amantadine dose × 1).

### Statistical analysis

Normally distributed variables are expressed as the means ± standard deviations and non-normally distributed variables as medians and interquartile ranges (IQRs). The SOL and FI data were analyzed after natural log transformation. Averages of the objective sleep measures over the measurement period were calculated. Inter-group comparisons of the means and medians were performed using unpaired *t*-tests and the Mann–Whitney U test, respectively, while categorical data were compared using the chi-square test. According to the light exposure, patients were divided into quartiles (lowest to highest, Q1–Q4), and associations among the variables were analyzed using linear regression models. Multivariable linear regression models comparing the objective sleep measures among the patients with PD according to light exposure were adjusted for age (per year), sex, BMI (per kg/m^2^), current smoking, drinking status (daily or not), education period (≥13 or < 13 years), household income (≥4 or < 4 million Japanese yen/year), hypertension, diabetes, use of sleeping drug agents, nocturnal void frequency (≥2 or < 2 times), bedtime (per minute), daytime physical activity (per count/min), daylength (per quartile), and Hoehn–Yahr stage (early or late). Statistical analyses were performed using Statistical Product and Service Solutions (version 24.0 for Windows; IBM Inc., Armonk, NY, USA). Two-sided *P*-values < 0.05 were considered statistically significant.

## Results

Although the mean age did not differ significantly between patients with PD and controls, the BMI, household income, daytime physical activity, measured day length, and habitual drinking and hypertension prevalence were significantly lower and nocturnal void frequency was significantly higher in the former than in the latter (Table 1). The median duration after PD diagnosis was 57 months (IQR, 33–102), and the mean daily levodopa equivalent dose was 470 ± 360 mg. Among the patients with PD, 116 had early-stage PD (stage I, *n* = 35; stage II, *n* = 81), and 73 had late-stage PD (stage III, *n* = 29; stage IV/V, *n* = 44).

**Table 1.** Basic and Clinical Characteristics Between Patients With PD and non-PD Controls

	Patients with PD	Non-PD control	
Characteristics	(*n* = 189)	(*n* = 1101)	*P*-value
*Basic parameters*			
Age, mean (SD), years	71.3 (7.6)	71.9 (7.1)	0.61
Gender, male	101 (53.4%)	515 (46.8%)	0.09
Body mass index, mean (SD), kg/m^2^	22.1 (3.6)	23.1 (3.1)	<0.001
Current smoker, number	9 (4.8%)	55 (5.0%)	0.89
Habitual drinker, number	25 (13.2%)	266 (24.2%)	0.001
Past education (13 years or more), number	54 (28.6%)	294 (26.7%)	0.59
Household income (4 million JPY or more), number	59 (31.2%)	437 (43.0%)	0.038
*Clinical parameters*			
Hypertension, number	65 (34.4%)	489 (44.4%)	0.016
Diabetes, number	23 (12.5%)	105 (9.5%)	0.21
Sleep medication, number	27 (14.3%)	118 (10.7%)	0.15
Nocturnal void frequency (two times or more), number	80 (42.8%)	324 (29.8%)	<0.001
Bedtime, mean (SD), clock time	22:19 (1:22)	22:28 (1:11)	0.11
Daytime physical activity, mean, counts/min	163.8 (90.3)	298.0 (102.2)	<0.001
Daylength, median (IQR), min	653 (623, 682)	678 (626, 769)	<0.001
*PD-related parameters*			
PD duration, median (IQR), month	57 (32, 102)	—	—
Hoehn–Yahr Stage (stage III or more), number	73 (38.6%)	—	—
Levodopa equivalent dose, mean (SD), mg/day	470.4 (360.0)	—	—

SD, standard deviation; IQR, interquartile range; PD, Parkinson’s disease; JPY, Japanese Yen.

Daytime light exposure was lower and nighttime light exposure was higher in patients with PD than in controls (Table 2). The daytime mean light intensities were 201.1 lux (IQR, 101.2–305.7) in patients with PD and 337.7 lux (IQR, 165.6–719.8) in controls (*p* < 0.001). The mean nighttime light intensities were 2.0 (IQR, 0.5–7.8) and 0.7 lux (IQR, 0.1–3.3) in the PD and control groups, respectively, (*p* < 0.001). Patients with PD had significantly less daytime exposure to ≥ 1000 lux light (median, 24.7 minutes vs. 50.5 minutes; *P* < 0.001) and significantly greater nighttime exposure to ≥ 10 lux light than the controls (median, 12.6 minutes vs. 5.5 minutes; *P* < 0.001). These associations were consistent in analyses stratified by gender and day length (Supplementary Table). In addition, all objective sleep measures, excluding SOL, were worse in patients with PD than in controls (mean SE, 70.8 ± 13.3% vs. 84.6 ± 7.7%, *p* < 0.001; mean WASO, 107.7 ± 58.7 minutes vs. 49.8 ± 29.1 minutes, *p* < 0.001; median SOL, 12.6 minutes (IQR, 6.4–19.7) vs. 18.5 minutes (IQR, 9.5–36.5), *P* < 0.001; median FI, 2.6 (IQR, 1.5–4.3) vs. 2.2 (IQR, 1.3–3.6), *P *= 0.006; mean total sleep time, 338.8 ± 99.7 minutes vs. 420.1 ± 69.6 minutes, *p* < 0.001). Sensitivity analysis regarding 2 days light data in patients with PD suggested that the median duration of daytime exposure to ≥ 1000 lux light in 2 days and 7 days was 26.0 minutes (IQR, 9.0–57.0) and 24.7 minutes (IQR, 8.8–45.7), respectively, which did not significantly differ (*p* = 0.66). The Spearman’s correlation coefficient of duration of daytime exposure to ≥ 1000 lux light between data in 2 days and 7 days in patients with PD was 0.86. Consistently, the median nighttime light intensities in 2 days and 7 days were 1.6 lux (IQR, 0.3–6.8) and 2.0 minutes (IQR, 0.5–7.8), respectively, which did not significantly differ (*p* = 0.24). The Spearman’s correlation coefficient of nighttime light intensity between data in 2 days and 7 days in patients with PD was 0.89. Furthermore, patients with PD had consistently and significantly less daytime exposure to ≥ 1000 lux light (median, 26.0 vs. 50.5 minutes; *p* < 0.001, respectively), and were exposed to greater nighttime light intensity (median, 1.6 vs. 0.7 lux; *p* < 0.001, respectively) than the controls.

**Table 2.** Light Exposure and Objective Sleep Measures Between Patients With PD and non-PD Controls

	Patients with PD	Non-PD control	
	(*n* = 189)	(*n* = 1101)	*P*-value
Light exposure parameters			
Mean light intensity, median (IQR), lux			
Daytime	201.1 (101.2, 305.7)	337.7 (165.6, 719.8)	<0.001
Nighttime	2.0 (0.5, 7.8)	0.7 (0.1, 3.3)	<0.001
Time above threshold, median (IQR), min			
Daytime exposure to 1000 lux or more	24.7 (9.3, 45.6)	50.5 (24.6, 95.9)	<0.001
Nighttime exposure to 10 lux or more	12.6 (2.0, 53.0)	5.5 (0, 25.5)	<0.001
Objective sleep measures			
SE, mean (SD), %	70.8 (13.3)	84.6 (7.7)	<0.001
WASO, mean (SD), min	107.7 (58.7)	49.8 (29.1)	<0.001
SOL, median (IQR), min	12.6 (6.4, 19.7)	18.5 (9.5, 36.5)	<0.001
FI, median (IQR)	2.6 (1.5, 4.3)	2.2 (1.3, 3.6)	0.006
TST, mean (SD), min	338.8 (99.7)	420.1 (69.6)	<0.001

SD, standard deviation; IQR, interquartile range; PD, Parkinson’s disease; SE, sleep efficiency; WASO, wake after sleep onset; SOL, sleep onset latency; FI, fragmentation index; TST, total sleep time.

Considering the PD stage, daytime light exposure was lower and nighttime light exposure was higher in patients with late-stage PD than in those with early-stage PD (Table 3). The mean daytime light intensities were 239.3 lux (IQR, 133.1–347.4) in patients with early-stage PD and 118.8 lux (IQR, 60.7–224.5) in patients with late-stage PD (*p* < 0.001). The mean nighttime light intensities in these groups were 1.1 (IQR, 0.3–3.4) and 3.4 lux (IQR, 1.1–12.7), respectively, (*p *= 0.001). The duration of daytime exposure to ≥ 1000 lux light was significantly shorter among patients with late-stage PD than among those with early-stage PD (median, 11.2 vs. 31.3 minutes; *p* < 0.001). Conversely, PD with late-stage patients had significantly less nighttime exposure to ≥ 10 lux light than patients with early-stage PD (median, 21.4 vs. 8.9 minutes; *p* = 0.016).

**Table 3.** Light Exposure Parameters Between Patients With Early and Late PD Stage

	PD stage		
	Early	Late	
	(*n* = 116)	(*n* = 73)	*P*-value
*Light exposure parameters*			
Mean light intensity, median (IQR), lux			
Daytime	239.3 (133.1, 347.4)	118.8 (60.7, 224.5)	<0.001
Nighttime	1.1 (0.3, 3.4)	3.4 (1.1, 12.7)	0.001
*Time above threshold, median (IQR), min*			
Daytime exposure to 1000 lux or more	31.3 (16.1, 53.1)	11.2 (4, 32.1)	<0.001
Nighttime exposure to 10 lux or more	8.9 (1.4, 47.1)	21.4 (6.4, 79.7)	0.016

IQR, interquartile range; PD, Parkinson’s disease.

**Figure 1.**
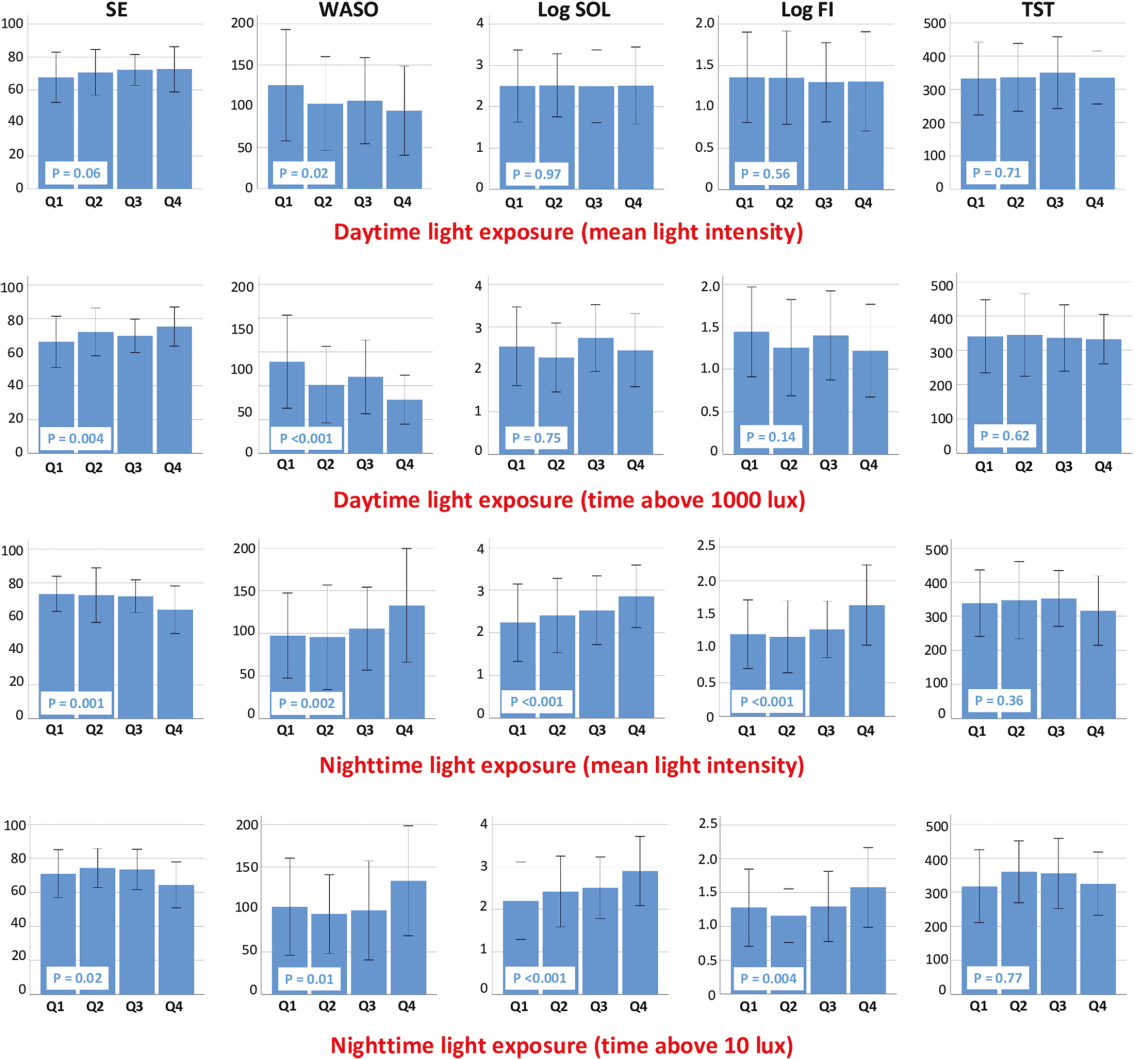
Associations of daytime and nighttime light exposure (lowest–highest, Q1–Q4) with objective sleep measures. Solid and error bars indicate means and standard deviations. *P*-values were calculated for the associations determined using linear regression analysis. SE, sleep efficiency; WASO, wake after sleep onset; SOL, sleep onset latency; FI, fragmentation index; TST, total sleep time.

Greater daytime light exposure was significantly associated with higher SE and shorter WASO (Figure 1), whereas greater nighttime light exposure was significantly associated with lower SE, longer WASO and SOL, and higher FI.

After adjustment for potential confounders, greater daytime exposure to ≥ 1000 lux light was significantly associated with higher SE and shorter WASO (*p* for trend = 0.018 and 0.011, respectively; Table 4). Compared with the shortest quartile group (Q1) of daytime exposure to ≥ 1000 lux light, SE was increased by 8.0% (95% confidence interval [CI]: 2.1 to 13.4; *p* = 0.008) and WASO was reduced by 36.9 minutes (95% CI: 13.4 to 60.3; *p* = 0.002) in the highest quartile group (Q4). During the nighttime, compared with Q1 of mean light intensity, Q4 had significantly lower SE by 6.8% (95% CI: 1.3 to 12.3; *p* = 0.016), longer WASO by 24.1 minutes (95% CI: 1.8 to 46.4; *p* = 0.034), longer SOL by 0.7 log min (95% CI: 0.3 to 1.0; *p* < 0.001), and higher FI by 0.3 log units (95% CI: 0.0 to 0.5; *p* = 0.006). Importantly, the multivariable model simultaneously including both daytime and nighttime light levels suggested independent associations of daytime and nighttime light exposure with SE and WASO (SE: *p* for trend = 0.027 and 0.039, respectively; WASO, *p* for trend = 0.016 and 0.031, respectively).

**Table 4.** Associations Between Light Exposure and Objective Sleep Measures in Patients With PD (*n* = 189)

	Daytime exposed time above 1000 lux, median [range], min					
	Q1	Q2	Q3	Q4		*R* ^2^
	4.3 [<9.7]	16.0 [9.7–24.7]	35.6 [24.7–45.5]	72.8 [>45.5]		
Adjusted^*^	(*n* = 48)	(*n* = 47)	(*n* = 47)	(*n* = 47)	*p*_trend_	
SE, mean (SE), %	67.3 (2.0)	71.3 (2.0)	69.5 (1.9)	75.2 (2.0)	0.018	0.165
Difference (95% CI)	ref	4.0 (−1.7, 9.7)	2.3 (−3.5, 8.0)	8.0 (2.1, 13.8)		
* p*		0.17	0.44	0.008		
WASO, mean (SE), min	124.2 (8.2)	102.3 (7.9)	116.7 (7.7)	87.3 (7.9)	0.011	0.303
Difference (95% CI)	ref	−21.9 (−44.7, 1.0)	−7.5 (−30.4, 15.5)	−36.9 (−60.3, −13.4)		
* p*		0.06	0.52	0.002		
SOL, mean (SE), log min	2.6 (0.1)	2.4 (0.1)	2.7 (0.1)	2.3 (0.1)	0.23	0.140
Difference (95% CI)	ref	−0.2 (−0.5, 0.2)	0.1 (−0.3, 0.5)	−0.3 (−0.7, 0.1)		
* p*		0.38	0.67	0.11		
FI, mean (SE), log unit	1.4 (0.1)	1.3 (0.1)	1.4 (0.1)	1.2 (0.1)	0.38	0.138
Difference (95% CI)	ref	−0.1 (−0.3, 0.2)	0.1 (−0.2, 0.3)	−0.1 (−0.4,0.2)		
* p*		0.60	0.67	0.24		
TST, mean (SE), min	327.9 (13.7)	333.2 (13.3)	343.2 (12.9)	351.6 (13.1)	0.20	0.345
Difference (95% CI)	ref	5.4 (−32.8, 43.6)	15.0 (−24.3, 53.5)	23.7 (−15.4, 62.9)		
* p*		0.78	0.44	0.23		
	LAN intensity, median [range], lux					
	Q1	Q2	Q3	Q4		*R* ^2^
	0.2 [<0.5]	1.0 [0.5–2.0]	4.1 [2.0–7.7]	20.4 [>7.7]		
Adjusted^*^	(*n* = 48)	(*n* = 47)	(*n* = 47)	(*n* = 47)	*p*_trend_	
SE, mean (SE), %	72.4 (1.9)	72.5 (1.9)	72.8 (1.9)	65.6 (1.9)	0.026	0.162
Difference (95% CI)	ref	0.1 (−5.2, 5.3)	0.4 (−4.9, 5.8)	−6.8 (−12.3, −1.3)		
* p*		0.98	0.88	0.016		
WASO, mean (SE), min	103.2 (7.7)	94.2 (7.7)	106.2 (7.8)	127.3 (7.8)	0.021	0.298
Difference (95% CI)	ref	−9.0 (−30.4, 12.4)	3.0 (−18.7, 24.7)	24.1 (1.8, 46.4)		
* p*		0.41	0.79	0.034		
SOL, mean (SE), log min	2.2 (0.1)	2.4 (0.1)	2.5 (0.1)	2.9 (0.1)	<0.001	0.196
Difference (95% CI)	ref	0.2 (−0.1, 0.6)	0.3 (−0.1, 0.6)	0.7 (0.3, 1.0)		
* p*		0.19	0.11	<0.001		
FI, mean (SE)	1.3 (0.1)	1.2 (0.1)	1.3 (0.1)	1.6 (0.1)	0.005	0.173
Difference (95% CI)	ref	−0.1 (−0.3, 0.1)	0.01 (−0.2, 0.2)	0.3 (0.1, 0.5)		
* p*		0.53	0.91	0.006		
TST, mean (SE), min	335.1 (12.6)	341.0 (12.8)	358.0 (12.8)	321.4 (12.8)	0.69	0.339
Difference (95% CI)	ref	5.9 (−29.2, 41.1)	22.9 (−12.9, 58.6)	−13.7 (−55.2, 15.9)		
* p*		0.74	0.21	0.46		

CI, confidence interval; SD, standard deviation; IQR, interquartile range; SE, sleep efficiency; WASO, wake after sleep onset; SOL, sleep onset latency; FI, fragmentation index; TST, total sleep time; LAN, light at night.

^*^Adjusted for age, gender, body mass index, current smoking, habitual drinking, past education, household income, hypertension, diabetes, sleep medication, nocturnal void frequency, bedtime, daytime physical activity, day length, and Hoehn–Yahr Stage.

## Discussion

The present study suggested that patients with PD have lower daytime light exposure and higher nighttime light exposure than older adults without PD, and among patients with PD, greater daytime light exposure and lower nighttime light exposure were significantly associated with better objective sleep measures independent of potential confounders, including daytime physical activity and disease stage. Importantly, daytime and nighttime light exposure was independently associated with objective sleep measures. To the best of our knowledge, such findings have not been reported previously.

Daily light exposure profiles in patients with PD were provided compared with those in non-PD older adults in the present study. PD is frequently accompanied by circadian misalignment, which is influenced by light exposure [2, 10]. Therefore, daily light exposure profiles are important behavioral features of patients with PD; however, daytime and nighttime light exposure among patients with PD in real-life situations has not been clarified. In our results, patients with PD were exposed to nearly half lower daytime light (median time ≥ 1000 lux, 24.7 vs. 50.5 minutes) and nearly three times higher nighttime light (mean light intensity, 2.0 vs. 0.7 lux) than non-PD older adults. In addition, late-stage patients with PD were exposed to nearly one-third lower daytime light (median time ≥ 1000 lux, 11.2 vs. 31.3 minutes) and nearly three times higher nighttime light (mean light intensity, 3.4 vs. 1.1 lux) than early-stage patients with PD. Although we suggested only average light data during daytime and nighttime in the present study, further investigations regarding most effective timing of light exposure, such as morning, midday, or evening, on sleep among patients with PD were needed.

Our findings suggested that higher daytime light exposure was significantly associated with better actigraphic sleep measures, although previous randomized controlled trials suggested no significant changes in objective sleep measures following bright light exposure. Although some studies recorded the effects of light therapy on subjective sleep, conflicting results were reported for subjective and objective sleep measures [12, 13, 23, 24]. In addition, objective sleep measures appear more suitable for quantitative analysis than subjective measures. A systematic review and meta-analysis of two randomized controlled trials suggested no significant changes in objective sleep quality following bright light exposure. One study of 31 American patients with PD suggested that exposure to 10 000 lux light significantly improved subjective sleep scores but not actigraphic sleep measures [12]. Another study including 18 Canadian patients with PD obtained similar results [13]. However, these studies provided intervention in the morning and/or evening, but evening exposure to bright light causes melatonin suppression and circadian phase delay, possibly resulting in decreased sleep quality [14, 15]. Thus, the present study is the first to find that higher daytime light exposure is significantly associated with better objective sleep measures in patients with PD. To better understand the effects of daytime light exposure on objective sleep measures, further interventional studies standing viewpoints of circadian physiology were warranted.

We demonstrated a significant association between indoor LAN and poor sleep quality in patients with PD for the first time, and the observed magnitude of influence might be significant. A recent systematic review indicated that most studies regarding LAN and sleep estimated outdoor LAN using satellite images, providing surrogate data of individuals’ LAN; thus, indoor LAN measured using light meters in the bedrooms was appropriate [6]. Recently, we reported the association between indoor LAN and sleep disturbances in 2947 Japanese adults [25]. To the best of our knowledge, only one clinical study reported an association between indoor LAN and poor actigraphic sleep measures in Japanese patients with bipolar disorders [26]. The present findings of lower SE in the highest LAN intensity quartile suggested a 14.5% higher risk of cardiovascular mortality when data from an 11-year prospective cohort study were considered [27].

Previous studies identified potential mechanisms underlying the associations of higher daytime and lower nighttime light exposure with better objective sleep measures. Light information crucially influences the circadian timing system in the SCN, and light exposure against the solar cycle can result in circadian misalignment [2]. Daytime bright light exposure increases nocturnal melatonin secretion, improving circadian alignment [5]. Conversely, LAN suppresses melatonin secretion, increases core body temperature, and stimulates brain activity [3, 4]. These effects of LAN on physiologic process were observed even at low light intensity (<10 lux) [28]. In our epidemiological studies, longer daytime exposure to ≥ 1000 lux light was significantly associated with higher melatonin levels [19]. In addition, LAN was significantly associated with poor objective sleep measures in a dose-dependent manner [29]. Interestingly, it was reported that LAN-induced dopamine neuron damage can trigger neuroinflammation and neurodegeneration [30]. In the present study, even patients with early-stage PD had lower daytime and higher nighttime light exposure than controls. In fact, we previously reported poor objective sleep quality in patients with early-stage PD [31]. Further interventional studies evaluating the mechanisms underlying the association between light exposure and sleep quality are needed.

The strengths of this study include the objective measurement of daily light exposure and sleep parameters in a large cohort comprising patients with PD and controls. However, this study has several limitations. First, since the cross-sectional design precluded causal inferences, additional longitudinal studies investigating the light exposure effects on objective sleep quality in patients with PD are warranted. Second, although the sleep measures were determined using a method validated against polysomnography, significant wrist tremors, dyskinesia, and REM sleep behavior disorder may have influenced the actigraphic data. However, no analytical methods are currently available to compensate for these involuntary movements. Third, light exposure and sleep measures were assessed only for 2 days in the control group, which may have led to misclassification. A moderately high correlation has been reported for sleep data obtained over 2 nights and 14 nights [32]. Although day-to-day reproducibility of both daytime and nighttime mean light intensities were moderately correlated (*r* = 0.61–0.70) [19] and inter-seasonal reproducibility was fair (*r* = 0.45–0.47) [16], it is unclear whether individual’s light exposure patterns are fixed or not in the long-term. Furthermore, nighttime light data were measured using two different light meters, and these were not validated. Luminance sensitivity and accuracy were similar but visibility and measurable area may be different between the two light meters. Fourth, the control group was not randomly selected, possibly leading to selection bias, although the BMI and estimated glomerular filtration rate data were similar to those obtained from a nationwide survey [33]. In addition, this study was conducted within a Japanese population, so light exposure habits and sleep behaviors may be different from non-Japanese population. Further studies are needed among different races. Fifth, although Hoehn–Yahr stage and sleep medication were adjusted for in the analytic models, residual confounding effects, such as disease severity and sleep disorders, might exist. In addition, cataracts or prior cataract surgery that related to light reception were not adjusted in the models. Lastly, the PD diagnosis was not supported by myocardial scintigraphy, albeit no prompt mistakes were observed during follow-up.

In conclusion, the present study suggested that patients with PD had lower daytime and higher nighttime light exposure than controls, and these findings were significantly associated with worse objective sleep measures independent of potential confounders, including daytime physical activity and disease stage. Importantly, independent associations of daytime and nighttime light exposure with objective sleep measures were detected. Although the present study treated light intensity, it would be important not only intensity but timing and wavelength when considering effect of light on sleep. Therefore, further studies are required regarding exposure to timing and wavelength of light in patients with PD. Also, further randomized trials are warranted to better understand the effects of daytime and nighttime light exposure on objective sleep measures.

## Supplementary Material

zsae036_suppl_Supplementary_Material

## Data Availability

The data sets generated and analyzed during the current study are not publicly available due no consent was obtained for publishing raw data but are available from the corresponding author on reasonable request.
