# Supplementary material for: Daily light exposure profiles and the association with objective sleep quality in patients with Parkinson’s disease: The PHASE study
Source: Sleep. 2024 Feb 8;47(8):zsae036. doi: 10.1093/sleep/zsae036 (PMC11321845; doi:10.1093/sleep/zsae036)
Supplement: zsae036_suppl_Supplementary_Material [file zsae036_suppl_supplementary_material.docx]

**Daily light exposure profiles and the association with objective sleep quality in patients with Parkinson’s disease: The PHASE study**

*Kenji Obayashi ^1*^; Keigo Saeki, ^1^; Yoshiaki Tai, ^1^; Yuki Yamagami, ^1^; Yuichi Esaki ^2^; Tadanobu Yoshikawa ^3^; Kazuma Sugie ^4^; Hiroshi Kataoka ^4^*

1. *Department of Epidemiology, Nara Medical University School of Medicine, Nara, Japan*
2. *Department of Psychiatry, Fujita Medical University School of Medicine, Aichi, Japan*
3. *Department of Ophthalmology, Nara Medical University School of Medicine, Nara, Japan*
4. *Department of Neurology, Nara Medical University School of Medicine, Nara, Japan*

* **Corresponding author**

Kenji Obayashi, MD, PhD

840 Shijocho, Kashiharashi, Nara, 634-8521, Japan

Department of Epidemiology, Nara Medical University School of Medicine, Nara, Japan

E-mail: obayashi@naramed-u.ac.jp

Phone: +81-744-29-8841, Fax: +81-744-29-0673
